# A botanical demonstration of the potential of linking data using unique identifiers for people

**DOI:** 10.1371/journal.pone.0261130

**Published:** 2021-12-14

**Authors:** Anton Güntsch, Quentin Groom, Marcus Ernst, Jörg Holetschek, Andreas Plank, Dominik Röpert, David Fichtmüller, David Peter Shorthouse, Roger Hyam, Mathias Dillen, Maarten Trekels, Elspeth Haston, Heimo Rainer

**Affiliations:** 1 Botanic Garden and Botanical Museum Berlin, Freie Universität Berlin, Berlin, Germany; 2 Biodiversity Informatics, Meise Botanic Garden, Meise, Belgium; 3 Biodiversity and Bioresources, Agriculture and Agri-Food Canada, Ottawa, Canada; 4 Major Floras, Royal Botanic Garden Edinburgh, Edinburgh, United Kingdom; 5 Department of Science, Royal Botanic Garden Edinburgh, Edinburgh, United Kingdom; 6 Division of Systematic and Evolutionary Botany, Universität Wien, Vienna, Austria; 7 Botany Department, Naturhistorisches Museum Wien, Vienna, Austria; Universidad Internacional de La Rioja, SPAIN

## Abstract

Natural history collection data available digitally on the web have so far only made limited use of the potential of semantic links among themselves and with cross-disciplinary resources. In a pilot study, botanical collections of the Consortium of European Taxonomic Facilities (CETAF) have therefore begun to semantically annotate their collection data, starting with data on people, and to link them via a central index system. As a result, it is now possible to query data on collectors across different collections and automatically link them to a variety of external resources. The system is being continuously developed and is already in production use in an international collection portal.

## Introduction

It is estimated that almost 400 million preserved plant specimens are stored in 3,324 herbaria worldwide and are available for a wide range of research activities [1]. Almost all of these specimens are mounted alongside a label on which various information is noted. The most commonly recorded details are the place, date of collection, the collector’s name, and the identification of the specimen. In the research process further information can be added in the form of annotations written directly on the mounting sheet or attached as separate labels. To improve access to specimens, multiple specimens are gathered in the same collection event, these are so called duplicates. These duplicates are then distributed and stored in different herbaria and consequently share their original label data.

In the course of digitization, herbaria worldwide share their digital specimen data and images with international biodiversity infrastructures such as the Global Biodiversity Information Facility (GBIF) [2] and the Biological Collection Access Service (BioCASe) [3], whose data portals provide simultaneous search and access to these distributed collection data. These infrastructures are based on data standards agreed by the community, such as Darwin Core [4] and ABCD (Access to Biological Collection Data) [5].

Although these collections are physically separated they are a global infrastructure that supports botanical and mycological research, particularly in taxonomy and evolution. One subset of these, known as type specimens, have been used to define all plant species and therefore underpin all botanical nomenclature [6]. Yet as a global infrastructure it is ill suited for easy access and the specimens are, more often than not, distant from their origin and the people who need them for research [7]. Only through digitization can these collections be made FAIR in the sense of Wilkinson et al. [8], but this also makes many demands on how these digital collections are presented.

The use of common data standards has led to great progress in harmonizing data elements, so that users and client software systems can now assume that data elements (e.g. “collector”, “country”, “altitude”) refer to the same semantic concept across collections. Nevertheless the data contents of these elements are still provided almost exclusively in the form of free text, so that linking and integration of data are difficult and in many cases impossible.

In the context of an initiative to establish a unified identifier system for natural history physical specimens (so called “CETAF Identifiers”) [9, 10], herbaria have begun to semantically annotate collection information to enable data integration between the collections themselves and with external resources. The identifiers are a necessary prerequisite for creating unambiguous and machine-readable references to and statements about specimens. The first step was the annotation of people who are documented as collectors of specimens in herbarium databases. People are a fundamental aspect of collections in a diverse range of roles, of which collector and determiner are arguably the most visible. However, they are also intrinsic to nomenclature as the authors of taxon names. The combination of collector name, collection number and date have been used for decades as identifiers for herbarium specimens, and are therefore key to being able to bring together duplicates held in different herbaria. People and their stories are also critical to engaging a wider audience for our collections. As globally available resources for people and person IDs, Wikidata [11], the Virtual International Authority File (VIAF) [12], and the Harvard University Herbaria Index of Botanists (HUH) [13] were primarily used. Together they have a high coverage, particularly for historical botanists [14]. The focus was initially placed on the annotation of person data because they were already available. The still expanding number of biographical records in Wikidata allows us to readily demonstrate how the use of jointly developed resources can contribute to the data quality of decentrally curated collections. Other data areas, such as geography and scientific names, will be addressed at a later stage.

The annotated specimen data are made freely available via RDF (Resource Description Framework) [15] interfaces of the participating institutions. For this purpose, best practices were jointly developed to harmonize the different implementations and the RDF formats used [16]. However, simply making annotated data available will not, in itself, stimulate their use and integration into information infrastructures or broaden the pool of data providers. Therefore, here we present a pilot study to show how semantic annotation can be used to transform previously disparate collections into a consistent information space and link them to other resources across disciplines.

The study does not primarily target the development and provision of a new resource of annotated collection data for automated integration into semantic web applications. Rather, it aims to show how a variety of resources can be automatically queried and summarized through comparatively simple annotation of collection data. For the respective queries, Linked Data techniques such as SPARQL and RDF are used in some cases, but in other cases also the specific APIs of the integrated information systems.

We provide landmarks for collections that want to establish semantic annotation as part of their curation strategy and infrastructures that want to incorporate linked biodiversity information into their data management and data publishing processes.

By linking locally curated data to robust globally available resources, this can create a shared data space that allows for precise cross-collection queries and data integration with external data resources. In our study, we demonstrate methods, feasibility, and benefits using annotation of collectors as an example. Transfer and extension to other data domains (e.g., localities) has already begun and will open additional query and inference possibilities.

## Methods

### Basic annotation process

To demonstrate the potential of semantic linking of botanical collection data, it was first necessary for the participating collections to annotate their specimen data with references to semantic resources. It was decided to start with the annotation of collector names, as the traditionally established system in botany of distributing duplicates to different collections was seen as having great potential for new cross-collection linkages. In addition, manifold external semantic resources on people exist that can be used for automated linkage of additional information. Annotations were either entered directly into the collection databases or prepared in bulk first by using spreadsheets such as MS Excel or OpenRefine and then imported.

To avoid conflicts with data protection regulations, no entries were created in particular for living people who do not already have pages in Wikidata, for example. We trust that only very few scientists will have no representation in e.g. Wikidata or ORCID in the future, due to their publication activities. Since essentially only existing information is linked, no significant additional data volumes are generated by the annotation activities. The discussion as to whether and to what extent a communitization of natural history collection data as a whole would be useful is currently being conducted in the community [17].

The enrichment process was performed by each contributing institution separately using their respective collection data management system. Initially, lists were generated of all different person records that could be found for specimens. These lists were subsequently processed by technicians experienced with the collection, starting with the most frequent people mentioned on specimens. In total, 3989 of 126956 distinct person name strings were annotated in the databases of the participating collections. Of the approx. 3.2 million specimen records with collector information, 1.45 million specimens could be enriched with person IDs in this way.

Initially, there were no specifications regarding the semantic resources to be used for annotation. Therefore, mostly one or more of the person identifiers from, for example, Wikidata, VIAF or HUH were used. In the course of the work, however, a preference developed for the use of Wikidata as the primary resource. This is partly because Wikidata has the largest and most dynamically evolving body of person data, but also because Wikidata has rich information on corresponding identifiers in other systems.

Automated methods of matching were also employed. Known information about the person records to be enriched, including their name(s), but also dates of birth and collection dates of the specimens, were used to construct algorithms consulting APIs (Application Programming Interfaces) of various resources of person identifiers and obtain probable matches.

Finally, the annotated collection data were published in a machine-readable way via RDF interfaces of the participating collections, so that they are accessible for harvesting and inference processes. Standards for uniform publication of the data have been developed in the context of CETAF technical working groups and are available via a ‘Stable Identifier Guide’ website [18].

### Central components and data harvesting

**Fig 1:**
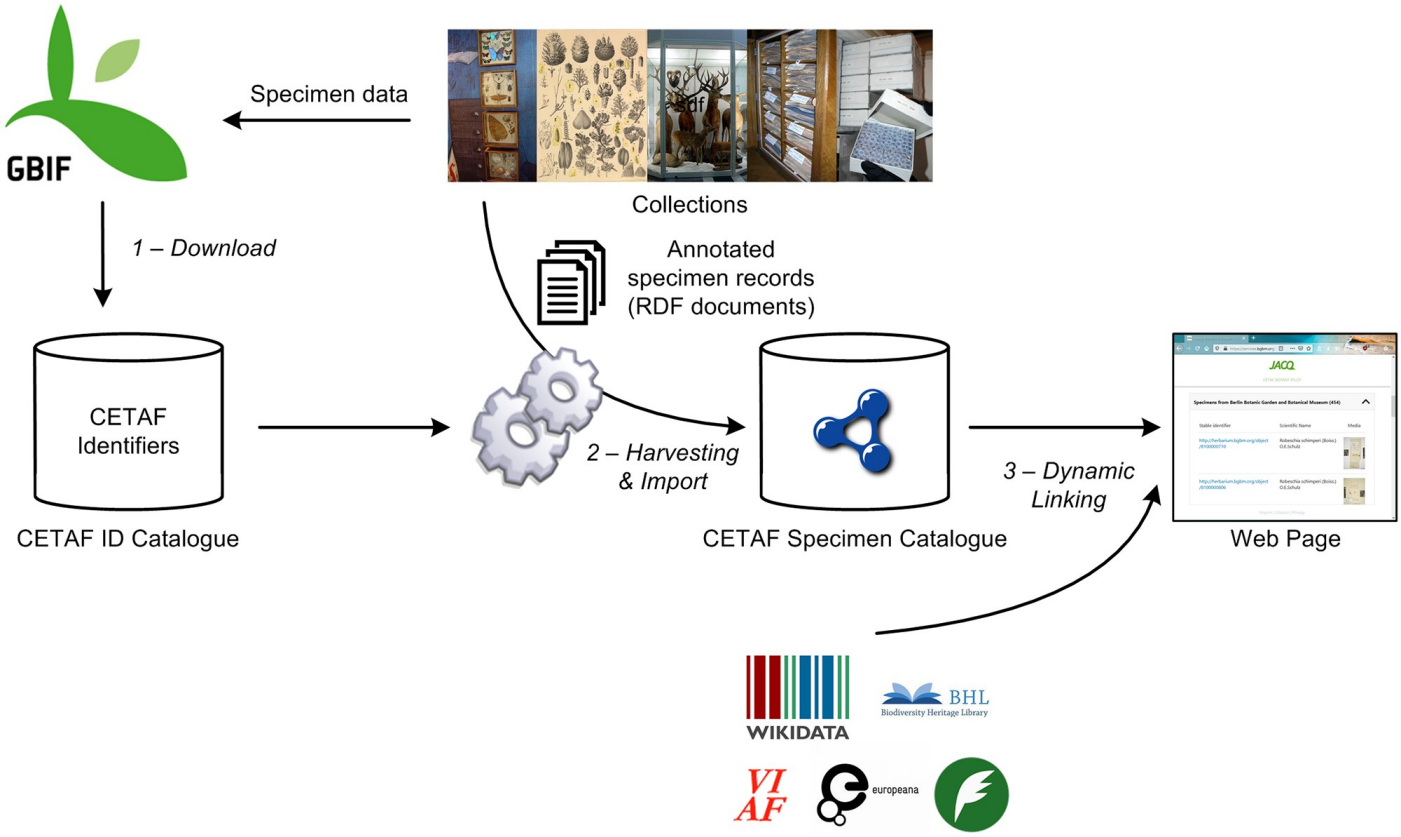
Pilot study architecture. GBIF provides a list of the currently available CETAF Specimen IDs (step 1), which are then used to harvest and import the corresponding specimen data of the collections into an RDF triple store (step 2). This provides the anchor point for generating dynamic web pages for people (step 3).

The core component of the system is a central index of specimens (the so called ‘CETAF Specimen Catalogue’) [19] which merges semantically annotated specimen data from participating institutions into a single RDF triple store, which is then updated regularly from the original data providers (Fig 1).

For specimens to be included into the Specimen Catalogue, institutions need to share their collections with GBIF, using a CETAF Identifier for their records (technically as a globally unique identifier in the occurrenceID field). By doing this, the GBIF data portal can be used to discover these identifiers and serves as a starting point for the Specimen Catalogue. In regular intervals, specimens published on GBIF are being downloaded as a snapshot. Specimen identifiers that comply with the syntax of CETAF Specimen IDs are extracted and imported into a temporary database, the CETAF Identifier Catalogue (left in the figure). This list of identifiers (20.9m as of October 26th 2020) is used by a harvester to retrieve specimen records from the source institutions into a semantic database, the CETAF Specimen Catalogue. As of October 26th 2020, this catalogue stores 16.4m specimen records from five institutions.

Ahead of importing into the index database, all harvested data are checked for technical validity (e.g. is the RDF technically valid, encoding of URIs, unicode encoding issues etc.) and secondly for having a standardized institution identifier. Validation for deeper nesting integrity of triple data is not done so far, here it would need efforts to check specific terms expecting linked nested triple data or to guess and check for meaningful possible data relations.

## Results

**Fig 2:**
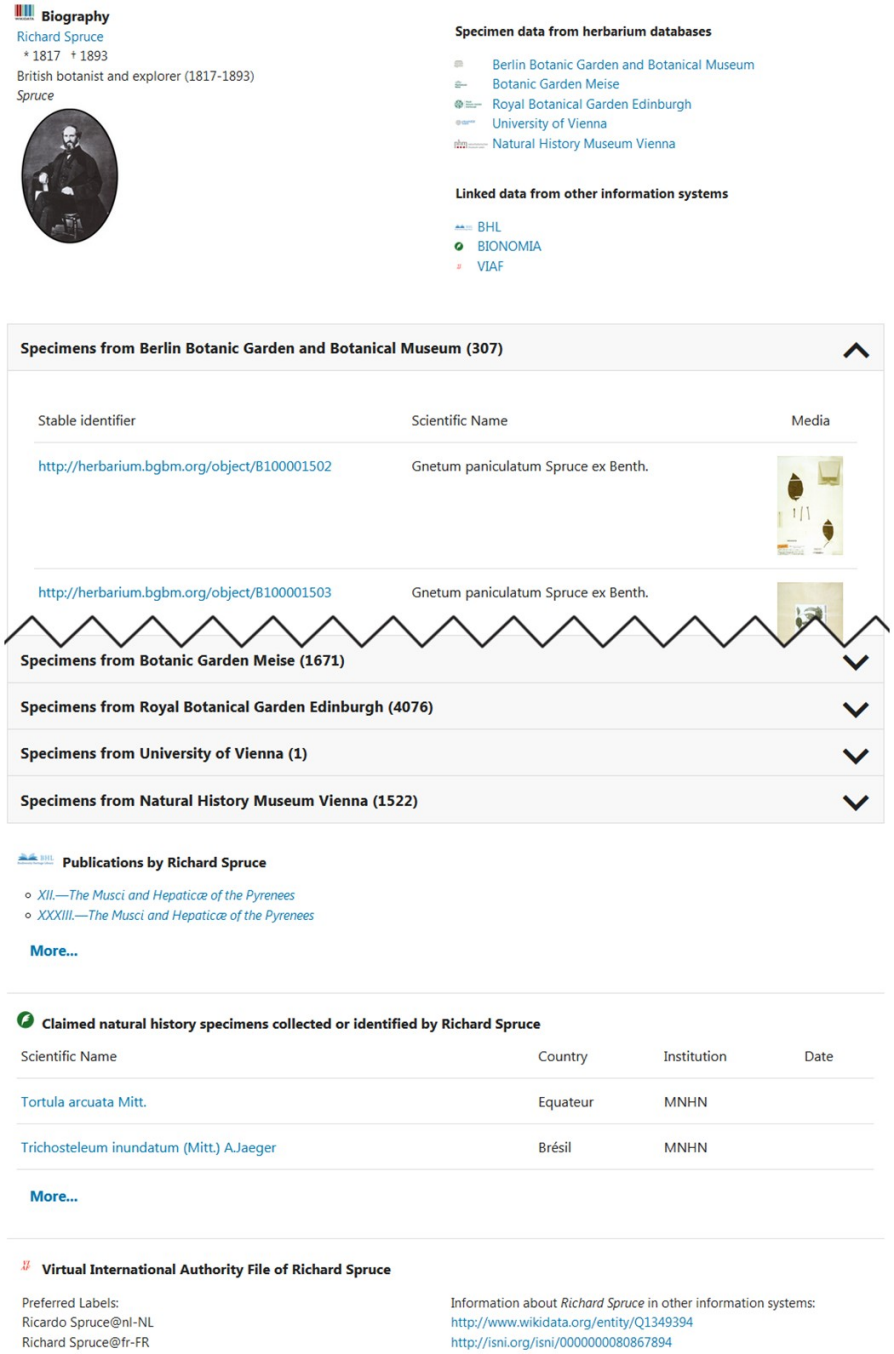
A dynamically created website for the British botanist Richard Spruce (1817–1893). Among other information, data on specimens from herbaria in Berlin, Meise, Edinburgh and Vienna (University and Natural History Museum) are compiled from semantic resources and made uniformly accessible [20].

In order to demonstrate the integration of semantic resources linked to people, a dynamic information service in the form of a website was implemented, which for a given Wikidata ID of a person pulls together information available for this person from a broad range of semantic sources and displays them in tabular form (Fig 2).

Specifically, the following sources were linked to the system:

### Specimens

Specimen data published semantically and annotated via RDF interfaces of the participating collections are transferred to a central index database via a harvesting process. This was connected to the pilot via a standardized SPARQL [21] query interface. By this method it was possible for the first time to investigate precisely how many specimens in different collections were collected by the same persons (Table 1).

**Table 1:** Overlap of collectors across collections.

	University of Vienna	Royal Botanical Garden Edinburgh	Meise Botanic Garden	JACQ collections
**Botanic Garden and Botanical Museum Berlin**	534 (55,972)	25 (59,062)	417 (356,987)	1,205 (164,369)
**JACQ collections**	499 (137,571)	22 (60,767)	316 (222,197)	-
**Meise Botanic Garden**	154 (90,518)	14 (28,550)	-	
**Royal Botanical Garden Edinburgh**	13 (29,789)	-		

Number of collectors with specimens in each two collections and (in brackets) number of corresponding specimens. Only digitized specimens were considered. The numbers for the herbarium network JACQ [22] exclude specimens from the Botanic Garden Berlin and the University of Vienna, which have been calculated separately.

### Wikidata

Wikidata plays a special and central role in linking people in collection data with many different resources. Firstly, Wikidata is the key resource for biographical data, which therefore no longer needs to be collected locally. In addition, Wikidata offers a variety of assignments of Wikidata IDs to identifiers used in other information systems. These include BHL, Europeana, VIAF and others. These links allow one to effectively combine person information from different information systems. In many cases, Wikidata users can even merge identifiers if there are several entries with different identifiers for the same persons into a single resource. Wikidata’s open editing model means that as long as primary references to biographical details can be found then an identifier can be minted for anyone with a minimal notability recruitment. The Botany Pilot uses Wikidata’s SPARQL endpoint for the queries to obtain the information as described above. For this purpose, it employs either the QID entered on the search page or the one found in our RDF store as a synonym for the entered ID.

### Bionomia

Bionomia [23] is a relatively new project, launched in October 2018. Its purpose is two-fold: (1) to give credit and valorize the living and deceased people that have spent time and effort collecting and determining the taxonomic identity of natural history specimens and, (2) to provide an open, public curation environment to build the associations between people and specimens. It makes use of information about people in ORCID and Wikidata and information about specimens from GBIF. As GBIF data are continuously being added to and amended it is refreshed wholesale every two weeks. Bionomia shares links between people and specimen records as JSON-LD [24] documents using elements from schema.org [25] and W3C Activity Streams. These linked data documents [26] are harvested for presentation in the pilot by using the collector’s Bionomia ID found in Wikidata.

### VIAF

The Virtual International Authority File (VIAF) combines multiple name authority files into a single name authority service by linking national-level authority records. This service allows researchers to identify names, locations, and works, while preserving regional variations in preferred language, spelling, and script. Linking bibliographic data is done using the VIAF ID provided by Wikidata or by the CETAF collections and accessed and integrated via an open API provided by VIAF. These data are retrieved as an RDF/XML file which is then processed in the tool as an RDF graph and searched for information on language-specific name variations as well as collector-related links to other information systems (e.g. DBpedia [27]).

### Biodiversity Heritage Library

The Biodiversity Heritage Library (BHL) [28] is an open access digital library for biodiversity literature and archives [29]. Over the last 14 years over 58 million pages, from the 15^th^ to 21st centuries have been digitized and made accessible via stable URLs and DOIs. BHL indexes the taxonomic names throughout the corpus, allowing researchers to locate publications about specific taxa. The publications offered by BHL are linked via the BHL creator IDs provided in Wikidata and are accessed and integrated using the REST-like BHL API. BHL provides democratized, global, online access to taxonomic literature that even during the pandemic has supported the disambiguation of people’s names and primary sources for biographies. Technically, the API provides the bibliographic data in JSON. The obtained files are searched for the collector’s publications, processing only a selection of information per publication for reasons of presentation.

### Europeana

Europeana [30] works with thousands of European archives, libraries and museums to share cultural heritage for enjoyment, education and research. It provides access to millions of books, music, artworks and more [31]. As of January 2021, 25 institutions supply data and images for 9.8 million natural history specimens to Europeana in the context of the OpenUp! initiative [32, 33]. The vast majority of the specimens are herbarium sheets. The classic Europeana portal uses person identifiers provided to link to their respective VIAF web pages [34]. Moreover, semantic concepts can be used to describe the type of object (e.g. illustration, heraldry or furniture), allowing a robust automated grouping of cultural items into collections [35].

Specimen images and other works offered by Europeana are linked via the Europeana person IDs retrieved from Wikidata and are accessed and integrated using the Europeana Search API that delivers JSON documents containing work-related information, such as media URLs, provider information and information about the location where the work is shown at.

### Availability of the query source code

The source code for querying the various sources and dynamically generating the corresponding person web pages is freely available via GitLab [36, 37].

### Trial deployment

With the dynamic information pages implemented in the project, which pull together information on the associated persons from various information systems for any Wikidata IDs, it is now possible to dynamically connect collection databases via these same Wikidata IDs. To demonstrate this functionality the system was successfully integrated into the collection information system JACQ, which is a shared herbarium data management system collaboratively maintained by more than 50 herbaria. Collectors are now linked to dynamically created person pages simply by using the annotation of their names with Wikidata IDs.

In order to be able to search for people independently of links provided in collection portals, an additional form has been made available in which it is possible to search for any person identifier and the information corresponding to it [38].

Collection data curators can now decide which data on persons should still be maintained locally in collection databases or whether preference should be given to the joint maintenance of person data as a whole by the community in Wikidata.

The system continues to grow by adding more resources and continuing the annotation activities in botanical collections.

## Discussion

The system presented here for integrating botanical collection data with linked data techniques shows, using person data as an example, that the geographic separation of natural history collections and the associated collection management systems can be overcome by the semantic annotation of data. This not only provides reliable links between specimens from identical collectors from different collections, but also a variety of integration possibilities with external and interdisciplinary information sources, for example biographies and literature. Making collection data more precise through annotation further provides an important contribution to advanced methods for detecting specimen duplicates across collection boundaries [39]. The pilot shows also that the effort of semantic annotation is reduced by strategic prioritization as well as the continuously improving annotation methods.

By integrating the new dynamic information service into the data portal of the JACQ collection management system, the results can be used directly by a broad user community.

Encouraged by these results, we now want to expand the technical basis to allow data access via an API. In this way, any collection portal can precisely query data on individuals and integrate it into their own websites and data integration workflows. Furthermore, it is planned for the API to deliver all collector data in an RDF serialization format. JSON-LD is suitable for this purpose, as it embeds linked data in the familiar JSON format. The endpoint will be accessible via a URL and will provide information about a collector by adding an identifier to the URL.

An improvement to the harvesting process could be achieved by the participating collection institutions by implementing a catalogue service that provides a plain list of all available specimen identifiers and possibly a simple classification (e.g. country and family). With this catalogue, aggregators could scan the available RDF resources before downloading and ignore records that may not be needed. The current detour via the use of the GBIF API could thus be avoided.

People’s identities provide a simple test cases for this pilot, because people are unitary entities and there is a wealth of biographical data available [14]. Nevertheless, parallel to the development of a platform-independent API, we want to extend the annotation activities thematically into other areas. The linking of free text information on collection sites with GeoNames [40] identifiers has already begun. It is expected that these activities will dramatically improve query and inference possibilities with geographic reference. Further possible topics are specimen citations in molecular sequences, literature and general terminology resources such as descriptions of habitats. A similar approach might also be used with taxonomic names given to specimens. However, this is more challenging to interpret than people, as the concept of a taxon and its given name are open to interpretation of the taxonomic authority.

Although the process of manual disambiguation of person names may seem inefficient, there are comparatively few highly productive collectors and identifiers so it is not necessary to connect a large number of person names to connect a large number of specimens. Furthermore, by pooling biographical details, particularly in Wikidata, the collections community benefits from a single biographical resource, rather than each building their own.

## Conclusion

The ability of natural history collections to link their datasets with external resources is an important prerequisite for the implementation of the Extended Specimen concept [41], as it is implemented, for example, within the DiSSCo initiative (Distributed System of Scientific Collections) [42]. In the long run, collection management systems must offer special functions that allow such links to be created and checked effectively. The approaches developed in this pilot study make an important contribution to this.

Nevertheless, annotation of textual data currently still involves a large proportion of disambiguation decisions made manually by data curators. This proportion can be reduced by applying specific patterns in the data that provide clear indications of semantic mappings to potentially many datasets rather than individually. Such methods are currently being tested experimentally in the project context of annotating geographic data.

## References

[1] Thiers B, The World’s Herbaria 2019: A summary report based on data from Index Herbariorum; 2020. http://sweetgum.nybg.org/science/ih/. [cited 2021 February 15].

[2] GBIF—Global Biodiversity Information Facility [Internet]. https://www.gbif.org/. [cited 2021 Jun 22].

[3] Biological Collection Access Service [Internet]. https://www.biocase.org/. [cited 2021 Jun 22].

[4] Wieczorek J, Bloom D, Guralnick R, Blum S, Döring M, Giovanni R, et al. Darwin Core: An Evolving Community-Developed Biodiversity Data Standard. PLoS ONE 2012;7(1):e29715. doi: 10.1371/journal.pone.0029715 22238640 PMC3253084

[5] Holetschek J, Dröge G, Güntsch A, Berendsohn WG, The ABCD of primary biodiversity data access. Plant Biosystems 2012;146(4). doi: 10.1080/11263504.2012.740085

[6] Turland NJ, Wiersema JH, Barrie FR, Greuter W., Hawksworth DL, Herendeen PS, et al. (eds.). International Code of Nomenclature for algae, fungi, and plants (Shenzhen Code) adopted by the Nineteenth International Botanical Congress Shenzhen, China, July 2017. Regnum Vegetabile 2018;159. doi: 10.12705/Code.2018

[7] López A, Sassone AB, The Uses of Herbaria in Botanical Research. A Review Based on Evidence From Argentina. Front. Plant Sci. 2019;10:1363. doi: 10.3389/fpls.2019.01363 31787992 PMC6853993

[8] Wilkinson M, Dumontier M, Aalbersberg I, Appleton G, Axton M, Baak A, et al. The FAIR Guiding Principles for scientific data management and stewardship. Sci Data 2016;3(1):160018. doi: 10.1038/sdata.2016.18 26978244 PMC4792175

[9] Güntsch A, Hyam R, Hagedorn G, Chagnoux S, Röpert D, Casino A, et al., Actionable, long-term stable and semantic web compatible identifiers for access to biological collection objects. Database. 2017(1): bax003. doi: 10.1093/database/bax003 28365724 PMC5467547

[10] Groom Q, Hyam R, Güntsch A, Stable identifiers for collection specimens. Nature 2017;546, 33. doi: 10.1038/546033d 28569808

[11] Wikidata [Internet]. https://www.wikidata.org/wiki/Wikidata:Main_Page. [cited 2021 Jun 22].

[12] VIAF: The Virtual International Authority File [Internet]. http://viaf.org/. [cited 2021 Jun 22].

[13] Harvard University Herbarium—Index of Botanists [Internet]. https://kiki.huh.harvard.edu/databases/botanist_index.html. [cited 2021 Jun 22].

[14] Groom Q, Güntsch A, Huybrechts P, Kearney N, Leachman S, Nicolson N, et al. People are essential to linking biodiversity data. Database. 2020: baaa072. doi: 10.1093/database/baaa072 33439246 PMC7805432

[15] Resource Description Framework (RDF) [Internet]. https://www.w3.org/RDF/. [cited 2021 Jun 22].

[16] CETAF Specimen Preview Profile [Internet]. https://cetafidentifiers.biowikifarm.net/wiki/CETAF_Specimen_Preview_Profile_(CSPP)). [cited 2021 Jun 22].

[17] Fichtmueller D, Güntsch A, Paul D, Bourgoin T, Agosti D, Häffner E, Better Together: Merging our Knowledge About People, Places, Collections, and Taxonomies with Wikidata. SPNHC & ICOM NATHIST Virtual Conference 2020, 8–12 June 2020, Abstracts, https://spnhc.org/wp-content/uploads/2020/06/SPNHC_ICOM-NATHIST-2020-Abstracts.pdf

[18] CETAF Stable Identifier Guide [Internet]. https://cetafidentifiers.biowikifarm.net/wiki/Main_Page. [cited 2021 Jun 22]

[19] CETAF Specimen Catalogue [Internet]. https://cetafidentifiers.biowikifarm.net/wiki/CETAF_Specimen_Catalogue. [cited 2021 Jun 22].

[20] JACQ CETAF BOTANY PILOT—Richard Spruce [Internet]. https://services.bgbm.org/botanypilot/person/q/Q1349394. [cited 2021 Jun 22]

[21] SPARQL 1.1 Query Language [Internet]. https://www.w3.org/TR/sparql11-query/. [cited 2021 Jun 22].

[22] JACQ [Internet]. http://jacq.org/. [cited 2021 Jun 22].

[23] Bionomia [Internet]. https://bionomia.net/. [cited 2021 Jun 22].

[24] JSON for Linking Data [Internet]. https://json-ld.org/. [cited 2021 Jun 22].

[25] schema.org [Internet]. https://schema.org/. [cited 2021 Jun 22].

[26] Bionomia JSON-LD document for Richard Spruce [Internet]. https://bionomia.net/Q1349394/specimens.json [cited 2021 Jun 22].

[27] DBpedia—Global and Unified Access to Knowledge Graphs [Internet]. https://www.dbpedia.org/. [cited 2021 Jun 22].

[28] BHL—Biodiversity Heritage Library [Internet]. https://www.biodiversitylibrary.org/. [cited 2021 Jun 22].

[29] Rinaldo C, Norton C, BHL, The biodiversity heritage library: An expanding international collaboration, Nat. Prec. 2009. doi: 10.1038/npre.2009.3620.1

[30] Europeana [Internet]. https://www.europeana.eu/. [cited 2021 Jun 22].

[31] Purday J, Think culture: Europeana.eu from concept to construction. Bibliothek Forschung und Praxis 2009;33(2). doi: 10.1515/bfup.2009.018

[32] Berendsohn WG, Güntsch A, OpenUp! Creating a cross-domain pipeline for natural history data. ZooKeys 2012;209: 47–54. doi: 10.3897/zookeys.209.3179 22859877 PMC3406465

[33] Holetschek J, Baumann G, Koch G, Berendsohn WG, Natural History in Europeana—Accessing Scientific Collection Objects via LOD. In Garoufallou E, Subirats Coll I, Stellato A, Greenberg J (eds.). Metadata and Semantics Research. 10th International Conference, MTSR 2016, Göttingen, Germany, November 22–25, 2016, Proceedings, 223–234.

[34] Europeana page for a herbarium specimen provided by Botanic Garden Meise [Internet]. https://classic.europeana.eu/portal/en/record/198/1X139691.html [cited 2021 Jun 22].

[35] Hierarchical browsing of objects based on semantic concepts on object types in Europeana [Internet]. https://www.europeana.eu/en/collections/ [cited 2021 Jun 22].

[36] CETAF Botany Pilot GitLab [Internet]. https://git.bgbm.org/cetaf/botanypilot [cited 2021 Jun 22].

[37] CETAF Botany Pilot GitLab—requests and queries [Internet]. https://git.bgbm.org/cetaf/botanypilot/-/blob/master/queries.md [cited Jun 22].

[38] CETAF Botany Pilot Search [Internet]. https://services.bgbm.org/botanypilot/. [cited 2021 Jun 22].

[39] Nicolson N, Paton A, Phillips S, Tucker A, Specimens as Research Objects: Reconciliation Across Distributed Repositories to Enable Metadata Propagation IEEE 14th International Conference on e-Science (e-Science), Amsterdam, 2018, pp. 125–135.

[40] GeoNames [Internet]. http://www.geonames.org/. [cited 2021 Jun 22].

[41] Lendemer J, Thiers B, Monfils AK, Zaspel J, Ellwood ER, Bentley A, et al. The Extended Specimen Network: A Strategy to Enhance US Biodiversity Collections, Promote Research and Education. *BioScience* 2020;70(1). doi: 10.1093/biosci/biz140 PMC6956879 31949317

[42] Distributed System of Scientific Collections [Internet]. https://www.dissco.eu/. [cited 2021 Jun 22].

